# Incidence, risk factors and management of post cesarean section surgical site infection (SSI) in a tertiary hospital in Egypt: a five year retrospective study

**DOI:** 10.1186/s12884-021-04054-3

**Published:** 2021-09-18

**Authors:** Khaled Gomaa, Ahmed R. Abdelraheim, Saad El Gelany, Eissa M. Khalifa, Ayman M. Yousef, Heba Hassan

**Affiliations:** Minia Maternity and Children University Hospital, Obstetrics and Gynecology Department, Faculty of Medicine, Minia University, Minia, Egypt

**Keywords:** Surgical site infection, Cesarean section, Incidence, Risk factors, Minia

## Abstract

**Background:**

Surgical site infection (SSI) is one of the commonest complications following cesarean section (CS) with a reported incidence of 3–20%. SSI causes massive burdens on both the mother and the health care system. Moreover, it is associated with high maternal morbidity and mortality rate of up to 3%.

This study aims to determine the incidence, risk factors and management of SSI following CS in a tertiary hospital.

**Methods:**

This was an observational case control retrospective study which was conducted at Minia maternity university hospital, Egypt during the period from January 2013 to December 2017 (Five years). A total of 15,502 CSs were performed during the studied period, of these, 828 cases developed SSI following CS (SSI group). The control group included 1500 women underwent cesarean section without developing SSI. The medical records of both groups were reviewed regarding the sociodemographic and the clinical characteristics.

**Results:**

The incidence of SSI post-cesarean section was 5.34%. Significant risk factors for SSI were; chorioamnionitis (adjusted odds ratio (AOR) 4.51; 95% CI =3.12–6.18), premature rupture of membranes (PROM) (AOR 3.99; 95% CI =3.11–4.74), blood loss of > 1000 ml (AOR 2.21; 95% CI =1.62–3.09), emergency CS (AOR 2.16; 95% CI =1.61–2.51), duration of CS of > 1 h (AOR 2.12; 95% CI =1.67–2.79), no antenatal care (ANC) visits (AOR 2.05; 95% CI =1.66–2.37), duration of labor of ≥24 h (AOR 1.45; 95% CI =1.06–2.01), diabetes mellitus (DM) (AOR 1.37; 95% CI =1.02–2.1 3), obesity (AOR 1.34; 95% CI =0.95–1.84), high parity (AOR 1.27; 95% CI = 1.03–1.88), hypertension (AOR 1.19; 95% CI = 0.92–2.11) and gestational age of < 37 wks (AOR 1.12; 95% CI = 0.94–1.66). The mortality rate due to SSI was 1.33%.

**Conclusions:**

The obtained incidence of SSI post CS in our study is relatively lower than other previous studies from developing countries. The development of SSI is associated with many factors rather than one factor. Management of SSI is maninly medical but surgical approach may be needed in some cases.

**Registration:**

Local ethical committee (Registration number: MOBGYN0040).

## Background

Cesarean section (CS) is the commonest obstetric surgical procedure, its global rates (including both emergency and elective) are ranging from 5 to 20% and the rates continue to rise in both developed and developing countries [1] . In Egypt, a high rate of CS “51.8%” was recorded in 2015 [2, 3].

The Center for Disease Prevention and Control (CDC) defind Surgical Site Infection (SSI) as an infection which happens within a month following surgical intervention and includes three types: superficial incisional SSIs (primary & secondary), deep incisional SSIs (primary & secondary) and organ/space SSIs [3].

In the developing countries, SSI is the major infection affecting more than 60% of the operated patients [3]. In USA, SSIs are responsible for about 15% of all nosocomial infections [4].

Surgical site infections (SSIs) are significant cause of morbidity and mortality in patients performing all types of operations, these infections lead to an increase in the duration of hospitalization, costs of health care, morbidity and mortality. The maternal infectious morbidity increases to eight-folds after cesarean delivery compared to vaginal births [5] . Globally, a wide range of SSI rates after CS was reported, it has been reported that this rate ranged from 3 to 15% “depending on the methods used to identify infections [6] . Different rates of SSI post CS were reported in many countries; 19% in Kenya [7], 16.2% in Nigeria [8], 10.9% in Tanzania [9], and 9.7% in Vietnam [10]. Also, SSI complicated 14.4% of CSs in Jordan [11], 4.5% in Saudi Arabia [12], 6.2% in Turkey [13], 11% in Ethiopia [14], 10.9% in Rwanda [15], 12.6% in Nepal [16] and 24.3% in Pakistan [17]. However lower rates were reported in China “3.34%” [18] and Israel “3.7%” [19]. Meanwhile in another study, this rate was found in 48.2% of cesarean deliveries (CDs) at a referral centre in Tanzania [20]. These studies demonstrate that these wide differences in the overall SSI rates were attributed to many factors such as the study sample as well as preexisting diseases in addition to the reliable methods for SSI documentation.

Several risk factors for SSI post CS were identified. Of these, some preoperative conditions such as hypertension, Diabetes Mellitus (DM), obesity, high parity, prolonged labor, premature rupture of membranes (PROM), chorioamnionitis, emergency CS, and no antenatal care (ANC) visits [5, 14–16, 20–25].

Also, some intraoperative conditions were significantly associated with developing SSI such as prolonged operative duration, vertical skin incision in addition to interrupted skin suturing [14, 18, 20, 21, 24] . While, blood loss was found to be a risk factor for SSI postoperatively [5, 14, 23].

In general, infection (sepsis) in the postpartum period is considered one of the leading causes of maternal mortality and morbidity from which SSI shares the principal proportion [5, 26, 27]. The knowledge about the incidence and associated risk factors for SSI after CS helps to increase the awareness among the health care professionals for preventing SSI and improving maternal outcome. To the best of our knowledge, the incidence, risk factors and management of SSI among women undergoing CS in Egypt have not yet been fully investigated. Therefore, the objective of this study was to determine incidence, risk factors and management of SSI following CS at a tertiary hospital in Egypt over 5 years.

## Methods

### Study settings

This was a an observational case control retrospective study carried out at Minia maternity university hospital, Minia governorate, Egypt from January 2013 to December 2017 (5 years). The setting of the current study receives referrals from nine district hospitals and serves a population of 5.5 million people, and it is the only tertiary hospital in Minia Governorate, Egypt.

### Study population

In the current study there were 15,502 CSs from a total of 40,750 deliveries. Of these, 828 cases underwent CS and developed SSI following CS (SSI group). However, 1500 records of cases that underwent CS without developing SSI were randomly chosen (by simple randomization method) and considered as a control group. We included all patients who underwent CS and developed SSI during the study period. SSI was based on the definition proposed by CDC guidelines [28–30].

### Data collection

Data were collected from the medical records (including delivery and operating room records) using special proforma by a well-trained person. Records of cases with excessive missed data were excluded. Demographic, clinical characteristics and obstetric data for each case were recorded including age, parity, residence, anthropometric data, antenatal care, gestational age at delivery, medical complications such as diabetes mellitus, hypertension and anemia, occurrence of PROM or chorioamnionitis, duration of labor, duration of CS, blood loss and duration of hospital stay.

#### Peri-operative hospital protocol for reducing infectious morbidity associated with cesarean section

##### Antibiotic prophylaxis

One gram of broad spectrum antibiotic is given to all patients before skin incision.

##### Urinary catheterization

An indwelling urinary catheter is inserted under complete aseptic conditions and removed once the patient is mobile after the CS.

##### Skin preparation

Antiseptic solution (Povidone-Iodine 10%) is used for skin preparation before CS.

##### Vaginal preparation

Aqueous iodine vaginal preparation is used before CS in women with ruptured membranes to reduce the risk of endometritis.

### Post-operative hospital protocol and management of infected patients

The hospital protocol after CS is to schedule a post-operative follow up appointments for all patients one and 6 weeks after the CS in the outpatient clinic. Diagnosis of patients who developed SSI was usually detected at these appointments mostly at the first (1 week) appointment. Patients who suffered from symptoms suggestive of SSI before or after these appointments were self-referred to this clinic as per the post-operative instrcuctions explained in the discharge card given to each paient on discharge from the hospital.

Management of patients who developed SSI after CS was determined according to the type of infection (superficial incisional SSI, deep incisional SSI or organ/space SSI) and presence of comorbidities as DM. Patients with superficial incisional SSI were usually managed at the outpatient clinic with repeated dressings and antibiotics till either complete healing of the wound achieved or hospital admission needed. Patients with deep incisional SSI and organ/space SSI were admitted to the inpatient ward according to the hospital protocol for medical ± surgical management.

### Study outcomes

The main outcomes of the study were the incidence, risk factors, morbidity and mortality resulting from SSI.

### Ethical considerations

Minia Maternity hospital’s ethical committee approved the study (reference number: MOBGYN0040). All methods were performed in accordance with the relevant guidelines and regulations (Declaration of Helsinki). The ethical committee of the Department of Obstetrics and Gynaecology, Minia University Hospital waived the need of informed consent and the reasons for waiving the consent were that in addition to being a retrospective study, the research presents no more risk of harm to participants and involves no procedures for which written consent is normally required outside of the research context. Moreover, the patients’ confidentiality were not breached.

### Statistical analysis

Analysis was carried out using SPSS program (version 21). Mean and standard deviation (SD) were calculated for continuous variables and the incidence rate of SSI was calculated. T-test was used for the comparison between cases and control groups regarding continuous variables. However, Chi-square test and/or Fisher exact test were used for categorical variables. Odds ratio (OR) and 95% CI were calculated. For the determination of risk factors associated with SSI, univariate and multivariate logistic regression analyses were used.

## Results

**Fig. 1:**
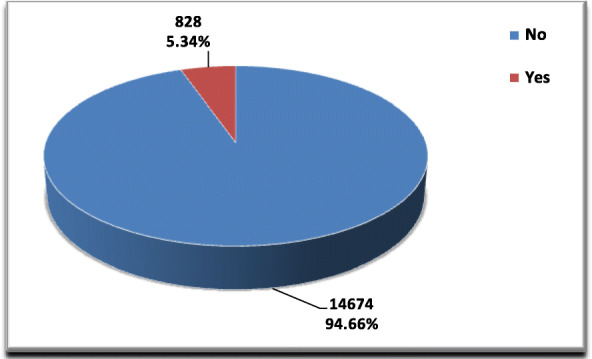
SSI among CS deliveries

A total of 15,502 CSs were performed during the studied period, of these, 828 cases developed SSI following CS. The overall incidence of SSI in our hospital during the study period was 5.34% (Fig. 1). The mean and the SD for cases that had SSI after CS was 5.92 ± 0.49 (3–25) days. The results revealed that there was no statistically significant difference between cases and control groups regarding age and residence. SSI group had a significantly higher number of cases with high parity (> 4) compared to control group (13.5 vs. 8.5%, *p* < 0.001). Also, the occurrence of preterm delivery (< 37 wks) was higher in SSI group (16.8% vs 11.40%, *p* < 0.001). Emergency CSs rate was significantly higher in SSI group (81.2% vs. 64.3%; *p* < 0.001, COR = 2.40). Also, we found that SSI was associated with longer duration of CS (> 1 h), prolonged labor (≥24 h) and high blood loss (> 1000 ml), (*P* < 0.001). Furthermore, there was a significant association between SSI and chorioamnionitis (16.8%, *p* ≤ 0.001, COR = 5.40), PROM (43.6%, *p* ≤ 0.001, COR = 4.93), no ANC visits (32.7%, *p* ≤ 0.001, COR = 2.33), obesity (16.4%, *p* ≤ 0.001, COR = 1.67), diabetes mellitus (10.0%, *p* ≤ 0.001, COR = 1.83) and hypertension (5.0%, *p* ≤ 0.016, COR = 1.68) (Table 1).

**Table 1:** Basic and obstetric charactersitcs of SSI and control groups

Variable	SSI group (***n*** = 828)	Control group (***n*** = 1500)	P. V (Sig.)	Crude Odds ratio (95% CI)
**Age (mean ± SD)**	31.20 ± 4.90	30.90 ± 4.80	0.15^NS^	–
< 19	95% CI (30.9–31.5) 87 (10.50%)	95% CI (30.7–31.1) 141 (9.40%)	0.54^NS^	1.12 (0.84–1.49)
20–34	678 (81.90%)	1231 (82.00%)		1.0
≥ 35	63 (7.60%)	128 (8.60%)		0.89 (0.65–1.23)
**Residence**				
Urban	361 (43.60%)	676 (45.10%)	0.50^NS^	1.0
Rural	467 (56.40%)	824 (54.90%)		1.06 (0.89–1.26)
**Parity**				
1–4	716 (86.50%)	1372 (91.50%)	**≤ 0.001**	1.0
> 4	112 (13.50%)	128 (8.50%)		1.68 (1.28–2.19)
**Gestational age**				
< 37 wks.	139 (16.80%)	172 (11.40%)	**≤ 0.001**	1.56 (1.22–1.98)
≥ 37 wks.	689 (83.20%)	1328 (88.60%)		1.0
**Type of the CS**				
Elective	156 (18.80%)	536 (35.70%)	**≤ 0.001**	1.0
Emergency	672 (81.20%)	964 (64.30%)		2.40 (1.95–2.94)
**Duration of CS**				
≤ 1 h	112 (13.50%)	421 (28.10%)	**≤ 0.001**	1.0
> 1 h	716 (86.50%)	1079 (71.90%)		2.49 (1.98–3.14)
**Bleeding (> 1000 ml)**				
No	739 (89.30%)	1433 (95.50%)	**≤ 0.001**	1.0
Yes	89 (10.70%)	67 (4.50%)		2.58 (1.85–3.58)
**Labour Duration**				
< 24 h	719 (86.80%)	1382 (92.10%)	**≤ 0.001**	1.0
≥ 24 h	109 (13.20%)	118 (7.90%)		1.78 (1.35–2.34)
**Chorioamnionitis**				
No	689 (83.20%)	1446 (96.40%)	**≤ 0.001**	1.0
Yes	139 (16.80%)	54 (3.60%)		5.40 (3.89–7.49)
**PROM**				
No	467 (56.40%)	1297 (86.50%)	**≤ 0.001**	1.0
Yes	361 (43.60%)	203 (13.50%)		4.93 (4.03–6.04)
**ANC visits**				
No	271 (32.70%)	296 (19.70%)	**≤ 0.001**	2.33 (1.86–2.91)
1–4	319 (38.50%)	599 (39.90%)	**0.003**	1.35 (1.11–1.66)
> 4	238 (28.80%)	605 (40.40%)		1.0
**Obesity**				
No	692 (83.60%)	1342 (89.50%)	**≤ 0.001**	1.0
Yes	136 (16.40%)	158 (10.50%)		1.67 (1.30–2.14)
**Diabetes Mellitus**				
No	745 (90.00%)	1414 (94.30%)	**≤ 0.001**	1.0
Yes	83 (10.00%)	86 (5.70%)		1.83 (1.34–2.51)
**Hypertension**				
No	787 (95.00%)	1455 (97.00%)	**0.016**	1.0
Yes	41 (5.00%)	45 (3.00%)		1.68 (1.09–2.59)

*NS* Not significant *P* value

*Significant *P* value

**Highly significant *P* value

The results of the multivariate analysis of risk factors for SSI are shown in Table 2. Several significant statistically important risk factors for SSI were found in this study, but varied in power. The risk factors for SSI were; chorioamnionitis (AOR 4.51; 95% CI =3.12–6.18; *p* ≤ 0.001), PROM (AOR 3.99; 95% CI =3.11–4.74; *p* ≤ 0.001), bleeding of > 1000 ml (AOR 2.21; 95% CI =1.62–3.09; *p* = 0.011), emergency CS (AOR 2.16; 95% CI =1.61–2.51; *p* = 0.012), duration of CS of > 1 h (AOR 2.12; 95% CI =1.67–2.79; *p* = 0.021), no antenatal care visits (AOR 2.05; 95% CI =1.66–2.37; *p* = 0.011), duration of labor of ≥24 h. (AOR 1.45; 95% CI =1.06–2.01; *p* = 0.038), DM (AOR 1.37; 95% CI =1.02–2.13; *p* = 0.011), obesity (AOR 1.34; 95% CI =0.95–1.84; *p* = 0.027), high parity (AOR 1.27; 95% CI = 1.03–1.88; *p* = 0.031), hypertension (AOR 1.19; 95% CI = 0.92–2.11; *p* = 0.020) and gestational age <  37 wks. (AOR 1.12; 95% CI = 0.94–1.66; *p* = 0.039).

**Table 2:** Multivariate analysis of risk factors for SSI

Variables	Adjusted Odds ratio (AOR) (95% CI)	P. V (Sig.)
**Age (≥ 35 years)**	0.66 (0.51–1.02)	0.312^NS^
**Parity (> 4)**	1.27 (1.03–1.88)	0.031*
**Gestational age (< 37 wks.)**	1.12 (0.94–1.66)	0.039*
**Type of the CS (Emergency)**	2.16 (1.62–2.51)	0.012*
**Duration of CS (> 1 h)**	2.12 (1.67–2.79)	0.021*
**Bleeding of (> 1000 ml)**	2.21 (1.62–3.09)	0.011*
**Prolonged labour (≥24 h.)**	1.45 (1.06–2.01)	0.038*
**Chorioamnionitis**	4.51 (3.12–6.18)	≤0.001**
**PROM**	3.99 (3.11–4.74)	≤0.001**
**No ANC visits**	2.05 (1.66–2.37)	0.001**
**Obesity**	1.34 (0.95–1.84)	0.027*
**Diabetes Mellitus**	1.37 (1.02–2.13)	0.011*
**Hypertension**	1.19 (0.92–2.11)	0.020*

*NS* Not significant *P* value

*Significant *P* value

**Highly significant *P* value

Table 3 shows types and management of SSI at our institution.

**Table 3:** Types and management of SSI

	Number (%) of cases (***N*** = 828)
**Types of SSI**	
Superficial incisional SSI	390 (47.10%)
Deep incisional SSI	232 (28%)
Organ/space SSI	206 (24.9%)
**Management of SSI**	
Medical (dressings and antibiotics)	521 (62.90%)
Surgical exploration	182 (22%)
Missing	125 (15.10%)

**Fig. 2:**
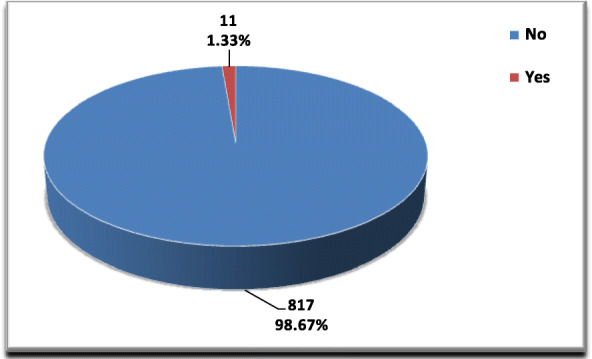
Mortality among SSI group

In the present study, 11 cases of the SSI group died accounting a rate of (1.33%) (Fig. 2).

## Discussion

In Egypt, the number of cesarean sections performed has recently doubled [22]. Surgical site infection following CS is one of the most common obstetric complications, it causes massive burdens on both the mother and the health care system and it is associated with high morbidity and mortality. It has been reported that the incidence of SSI after CS varied between 3 and 15% [6]. Unfortunately, very few data are available about the incidence, related risk factors and management of SSI after CS for the egyptian population. So, this study evaluated the incidence of SSI after cesarean sections, its associated risk factors and management in a tertiary referral hospital.

In the present study, the incidence of SSI following cesarean section was 5.34%. This incidence was comparable with a previous study in Egypt “conducted in Ain Shams tertiary referral hospital” which was 5.3% [22] . Also, this rate was in line with previous studies in Pennsylvania “6.5%” [21], Thai-Myanmar “5.9” [31], Ankara “6.2%” [13] and sub-Saharan Africa “7.3%” [32]. However, our obtained incidence was lower when compared with an incidence of 10.9% in Tanzania [9], 10.9% in Rwanda [15], 11% in Ethiopia [14], 16.2% in Nigeria [8], 19% in Kenya [7], 12.6% in Nepal [16] and 24.3% in Pakistan [17]. In contrast, our incidence was relatively high compared to previous studies in China “3.34%” [18] and Israel “3.7%” [19]. This difference might be attributed to the difference in the quality of both surgical care provisions and service among countries. Also, the differences between studies in sample size, settings and duration play an important role in these variations.

In the present study, no statistically significant difference was found between cases and controls regarding age and residence. These results agreed with earlier studies [5, 15]. While Borg et al., found controversial results, they found that SSI was significantly higher in rural regions due to less attention to infection control guidelines [22]. Also Kaye et al., reported that increased age was identified as a strong predictor for SSI [33].

Our results revealed that emergency CS, prolonged operative time (> 1 h) and prolonged duration of labor were significantly associated with SSI and were significant risk factors for SSI. Similarly, many authors reported that emergency CS was strongly correlated with high risk of infections [5, 21, 34], and this may be attributed to rapid preparation in response to maternal and/or fetal distress. In a recent study by Wodajo etal., they found that lenghthy CS duration (> 1 h) is a risk factor for SSI (AOR = 12.32, CI (5.5–27.8) [14]. Also, many studies found similar results [5, 9, 34]. Furthermore, a recent study in 2019 found that the duration of surgery of less than 1 h had a protective effect for prevention of SSI [24]. Exogenous contamination may be increased as a result of prolonged CS surgery which was responsible for developing infection [33]. Also, other explanations include anesthesia-related stress and extensive tissue trauma [24]. Recently, a study performed in Ethiopia found that prolonged labor was identified as independent risk factor for SSI (AOR = 3.48) [35]. Similar findings were reported by other studies [5, 36, 34]. In addition, other authors noticed similar observations related to prolonged duration of labor and the high incidence of SSI [34, 37].

Our results showed that blood loss was a significant risk factor for SSI (AOR 2.21; 95% CI =1.62–3.09; *p* = 0.011). Several studies reported that blood loss had a significant association with SSI developement [5, 14, 30, 38]. This may be attributed to hypoperfusion of the wound following anemia and the reduction of postoperative ambulation. In addition, blood loss and anemia cause a reduction in immunity and increase the risk of infection due to their negative effect on macrophages activity and impeding wound healing progress [39].

The current study demonstrated that there was significant assosiations between chorioamnionitis and PROM and develpement of SSI. Similarly, a recent study in Ehiopia found a significant association between chorioamnionitis and PROM and the development of SSI by almost four-folds [5]. Additionally, regarding chorioamnionitis, this result is consistent with previous studies conducted in both developed and developing countries such as China [18], Pennsylvania [21], Thailand [30] and Burkina Faso [40]. This may be explained by that the pathogens causing chorioamnionitis and complicated by metritis which acts as a nidus of infection to easily establish SSI. In addition, many studies support our findings and confirmed that SSI occurrence was significantly associated with PROM [36, 41]. Shrestha et al., in 2015 [16] reported that when the membrane ruptures, the amniotic fluid causes an infection due to its contact with the uterine and skin incisions.

Similar to our findings, there is a significant association between obesity and diabetes mellitus and the develpomenet of SSI following CS [17]. In addition, de Araújo et al. in 2014 [42] and Alfonso-Sanchez et al. in 2017 [43] reported a strong positive relationship between increased risk for SSI and anemia, hypertension, obesity and diabetes mellitus. Recently, Molla et al., in 2019 [5] found that cases with pregnancy-induced hypertension (PIH) were about five times more likely to develop SSI compared to normal ones. Similar studies confirmed the same findings [9, 19]. Pregnancy-induced hypertension had peripheral vasoconstrictive effect that causes hypoperfusion of the wound and cases complicated with PIH might have odema which may be responsible for further entry of organisms and development of the infection.

Many studies support our findings regarding the significant determinant risk factors for SSI post CS which were emergency CS, prolonged operative duration, prolonged labor, chorioamnionitis, PROM, high parity, significant blood loss, obesity, no antenatal care visits, hypertension and DM [5, 14–16, 20–25].

Regarding types of SSI encountered in our study, Approximately 50% of cases had superficial incisional SSI while 28% of cases developed deep incisional SSI and about one quarter of the cases had organ/space SSI. A similar study conducted at an Ethiopian referral hospital found that incisional SSI (superficial and deep types) developed in two third of cases while organ/space SSI developed in the remaining one third of cases. These findings are similar to ours [44].

Management of SSI in our study was medical (repeated dressings, antibiotics and follow up) in 521 cases (62.9%) and surgical in 182 cases (22%) while the data about management was missing in 125 cases (15.1%). The management in the Ethiopian study was similar to ours; medical management was performed in 57.1% of cases and surgical exploration was needed in 14.3% of cases and the data was missing in 28.6% of cases [44].

In the current study, 11 cases died from the SSI group accounting for a mortality rate of 1.33%. The mortality rate due to intra-abdominal infection following CS in our institution was 1.2% [45] which is very similar to the mortality due to SSI after CS reported in this study. In 2012 it was reported by Awad that SSI is associated with a mortality rate of 3% and about 75% of the deaths are directly attributable to SSI [46]. Moreover, Pearse et al., reported that patients developed SSI had a significantly increased mortality risk by 4:15 folds [47]. Recently, a mortality rate of 4.6% due to SSI after cesarean delivery was reported by Acosta et al., [48]. Furthermore, it has been reported that septicaemia after CS caused a maternal mortality rate of 10.7% [49].

The management challenges that we faced during the study were: pregnancy at extremes of reproductive life in about 20% of cases, more than 50% of patients were from deprived rural areas living away from the hospital, high parity, more than 80% of our patients had emergency CS with prolonged operative time more than 1 h, about 40% were poor antenatal care attendee, 32% were non-attendee, presence of many patients with risk factors as chorioamnionitis, PROM, obesity, hypertension and DM. Additional challenge was the loss of confidence felt by many patients towards the hospital and the medical team as a result of development of infection following CS in the hospital so we have to do extensive debriefing to convince them to continue their care at the same institution and more than 15% of them were managed outside our hospital.

Based on the findings of this study, the suggested modalities for reducing incidence and mortality from SSI after CS in our practice that can be adapted to similar environments include: strict adherence to hospital infection control policy and precautions for reducing infectious morbidity after CS, improving the general condition of the patients, addressing modifiable risk factors and encouraging regular ANC.

This study has some strengths. To the best of our knowledge, this is probably the first study from an Egyptian population about the incidence, risk factors and management of SSI after CS in a tertiary hospital. The study deals with an important health issue. The knowledge about the incidence and associated risk factors for SSI after CS helps to increase the awareness among the health care professionals for preventing SSI and improving maternal outcome. Furthermore, the relatively long study period (5 years) and the reasonably high number of participants are among the strength points. The limitations of the study include the retrospective design with absence of data about management in 15% of patients, lack of data about microbiological profile related to SSI, lack of data about the exact cause of death as we do not have a routine postmortem examination and being a single centre study. So, the generalizability may be limited. Additional limitation raised due to random selection of the control group which may cause a possible bias. Under-reporting of the incidence of SSI could constitute another cause of bias as some patients who developed SSI did not return to the hospital again. Further studies on a larger population and wide regions are needed to confirm our findings.

## Conclusions

In conclusion, the incidence of SSI post-cesarean section in our study was 5.34% which is relatively lower than other previous studies from developing countries. The development of SSI is associated with many factors rather than one factor. Significant risk factors for SSI were high parity, emergency CS, gestational age (< 37 wks.), prolonged labor (> 24 h), blood loss (> 1000 ml), prolonged operative time (> 1 h), chorioamnionitis, PROM, no ANC visits, obesity, diabetes and hypertensin. On the basis of our findings, targeting these risk factors could lead to better outcome of the CS surgery and is important in reducing the risk of developing SSI following CS.

## Data Availability

The datasets used and/or analyzed during the current study are available from the corresponding author on reasonable request.

## Abbreviations

CS: Cesarean Section
SSI: Surgical Site Infection
DM: Diabetes Mellitus
PROM: Premature Rupture of Membranes
ANC: Antenatal Care
COR: Crude Odds Ratio
AOR: Adjusted Odds Ratio
PIH: Pregnancy Induced Hypertension
